# Comparison of visceral, body fat indices and anthropometric measures in relation to chronic kidney disease among Chinese adults from a large scale cross-sectional study

**DOI:** 10.1186/s12882-018-0837-1

**Published:** 2018-02-17

**Authors:** Ying Dong, Zengwu Wang, Zuo Chen, Xin Wang, Linfeng Zhang, Jingyu Nie, Congyi Zheng, Jiali Wang, Lan Shao, Ye Tian, Runlin Gao

**Affiliations:** 1 Division of Prevention and Community Health, National Center for Cardiovascular Disease, Fuwai Hospital, Pecking Union Medical College & Chinese Academy of Medical Sciences, Beijing, 102308 China; 2 Fuwai Hospital, Pecking Union Medical College & Chinese Academy of Medical Sciences, Beijing, 100037 China

**Keywords:** Chronic kidney disease, Adiposity indices, Percentage body fat, Chinese adults, Cross-sectional study

## Abstract

**Background:**

The aim of the study was to assess the association between chronic kidney disease (CKD) and obesity in predicting CKD among Chinese adults, distinguishing between 5 different adiposity indices: visceral fat index (VFI), percentage body fat (PBF), body mass index (BMI), waist circumference (WC) and waist-to-height ratio (WHtR).

**Methods:**

A total of 29,516 participants aged 35 years or above were selected using a stratified multistage random sampling method across China during 2012–2015. CKD was defined as an estimated glomerular filtration (eGFR) < 60 ml/min/1.72m^2^.

**Results:**

The overall weighted prevalence of CKD was 3.94% (3.62% in males and 4.25% in females). All five adiposity indices had significant negative correlations to eGFR (*P* < 0.05). The area under the ROC (receiver operating characteristic) curves (AUC) for PBF was almost significantly larger than the other adiposity indices (*P* < 0.001). In addition, PBF yielded the highest Youden index in identifying CKD (male: 0.15; female: 0.20). In the logistic analysis, PBF had the highest crude odds ratios (ORs) in both males (OR: 1.819, 95% CI 1.559–2.123) and females (OR: 2.268, 95% CI 1.980–2.597). After adjusted for age, smoking status, alcohol use, education level, marital status, rural vs. urban area, geographic regions, and diagnosis of hypertension, diabetes mellitus, myocardial infarction and stroke, the ORs on PBF remained significant for both genders (*P* < 0.05).

**Conclusions:**

Obesity is associated with an increased risk of CKD. Furthermore, PBF was a better predictor for identifying CKD than other adiposity indices (BMI, WC, WHtR, and VFI).

**Electronic supplementary material:**

The online version of this article (10.1186/s12882-018-0837-1) contains supplementary material, which is available to authorized users.

## Background

Chronic kidney disease (CKD) has been a major global health problem. It also plays an important role in development of end-stage renal disease (ESRD) [1], all-cause mortality [2], and non-vascular health outcomes [3]. Currently, CKD is the 12th highest cause of death (14% of all deaths) worldwide [4]. A large representative survey in the United States has shown that the prevalence of CKD among adults aged ≥45 years’ was 11.6% [5]. In China, the overall prevalence of CKD among adults aged 18 years or older was 10.8% [6].

Obesity is becoming a global health concern. Over the past decade, changes in dietary and physical activity patterns has led to an increase in obesity prevalence, especially in large cities [7]. Previous studies have shown that the prevalence of obesity increased from 4.0% in 1993 to 10.7% in 2009 among Chinese adults [8]. An increasing body of evidence suggests that obesity, a risk factor of kidney disease, had a direct impact on the development of CKD and end stage renal disease (ESRD) [9]. Among many indicators of obesity, body mass index (BMI) and waist circumference (WC) have been widely used to define general and abdominal obesity, respectively [10]. However, BMI cannot differentiate between lean and fat mass, and WC does not account for the effect of height on risk [11]. Waist-to-height ratio (WHtR) was introduced as an alternative to WC. Additional obesity indicators include percentage body fat (PBF), which is defined as the percentage of individual fat mass over body weight [12]; and visceral fat index (VFI), an accurate and reliable indicator for evaluating body fat stored around important organs, such as the liver, intestines and pancreas [11].

The identification of CKD risk factors is essential, especially in the face of an increasing prevalence of CKD and obesity. Currently, it is unknown which of the existing adiposity indices is best for predicting CKD. Previous studies have only considered one or two types of adiposity measurement in relation to CKD [13–15]. Thus, we conducted this cross-sectional study in Chinese adults to assess the association between obesity and CKD, and compared the performance of five adiposity indices (BMI, WC, WHtR, PBF and VFI) to predict CKD.

## Methods

### Study population

This cross-sectional investigation was conducted from 2012 to 2015. This cross-sectional study was a part of a 2012–2015 survey on the prevalence of hypertension in China, spanning 31 provinces with 262 cities/counties; a detailed description of sampling methods has been previously published [13]. The 262 cities and counties were stratified into Eastern, Middle and Western regions, based on economic development status. Using simple random sampling (SRS), 16 cities and 17 counties were selected for this study, including 7 cities and 7 counties in the Eastern region, 6 cities and 6 counties in the Middle region, and 3 cities and 4 counties in the Western region. To meet the designed sample size of 35,000 participants aged ≥35 years and take non-response into account in the survey 56,000 subjects were randomly selected from the eligible sites. As a result, 34,994 participants completed the survey, the overall response rate was 62.5%. After excluded the subjects without blood pressure (*n* = 414), demographic information (*n* = 485), adiposity indices (*n* = 1836), laboratory test values (*n* = 2743), 29,516 were included in the final analysis. All the participants gave written informed consent prior to data collection. The ethical review committee of Fuwai Hospital (Beijing, China) and each participating center (Additional file 1) approved the study’s protocol.

### Adiposity indices

All staff involved in the study were trained and certified prior to survey implementation, according to a uniform protocol and operation manual. Height was measured to the nearest 5 mm with a standard stadiometer without shoes in the standing position. WC was measured to the nearest 5 mm directly touching the participant’s skin using a cloth tape. Weight was measured to the nearest 0.1 kg with light clothing and no shoes. PBF and VFI were measured using an OMRON body fat and weight measurement device (V-body HBF-371, OMRON, Kyoto, Japan). BMI was calculated as weight (in kilograms) divided by the square of height (in meters). WHtR was calculated as WC in centimeters divided by height in centimeters.

### Glomerular filtration rate assessment

Fasting blood samples were collected in the morning after 10-12 h fasting. The samples were processed properly and refrigerated immediately. All samples were analyzed in a designated central laboratory (Beijing Adicon Clinical Laboratories, INC, Beijing, China). The serum creatinine was measured using Jaffe’s kinetic method. Estimated glomerular filtration rate (eGFR) was calculated using the equation originating from the modified Modification of Diet in Renal Disease formula for Chinese patients [14]: eGFR = 175*Scr^-1.234^*age^-0.179^ [if female, *0.79], where Scr is serum creatinine concentration (mg/dL) and age is in years. CKD was defined as eGFR< 60 ml/min/1.73m^2^.

### Covariate measurements

Eligible participants completed the standardized questionnaire through face-to-face interview by trained staff to obtain information on demographic characteristics, lifestyle factors (i.e. smoking and alcohol use) and comorbidities (hypertension, diabetes mellitus, myocardial infarction and stroke). Smoking status was defined as a person who smoked in the past month or total cigarette consumption more than 20 packages in the lifetime. Alcohol use was defined as a person who consumed more than one alcoholic drink per week in the last 30 days. Hypertension was defined as systolic blood pressure (SBP) ≥ 140 mmHg, and/or diastolic blood pressure (DBP) ≥ 90 mmHg, and/or self-reported history of antihypertensive medications for treatment of hypertension in the last 2 weeks. Diabetes mellitus was defined as participants with previously diagnosed diabetes and used insulin or hypoglycemic agents, or had a fasting blood glucose ≥7.0 mmol/L.

### Statistical analysis

Continuous variables were presented as means ± standard deviations (SDs) and compared using the Student’s *t*-test or Wilcoxon rank-test. Categorical variables were presented as numbers (percentages) and compared using *Chi*-square test. The Pearson correlation coefficient was used to assess the correlation between each of the adiposity indices (BMI, WC, WHtR, PBF and VFI) and eGFR.

Receiver operating characteristics curve (ROC) analyses for both genders were used to determine the optimal cut-off values of each adiposity index for CKD with the maximum Youden index (sensitivity+specificity-1). Additionally, the areas under the ROC curve (AUCs) of these five adiposity indices were used to determine their reliability as predictive markers of CKD; statistical significance determined by applying the method of DeLong et al. [15] using MedCalc version 11.4.2.0.

Each adiposity index was divided into lower and higher level groups according to optimal gender- specific cut-off values performed in following ROC analyses. The association between the adiposity and CKD in both genders was calculated using multiple logistic regression analyses after adjusting age, education level, marital status, areas (rural/urban), region (eastern, middle and western region), smoking status, alcohol use, hypertension, diabetes mellitus, myocardial infarction and stroke.

The data analyses were performed using SAS version 9.3 (SAS Institute Inc., Cary, North Carolina, USA). Sample weights were adjusted for non-response, total population (according to 2010 Chinese census) and region to obtain Chinese nationally representative prevalence estimates. All *P* values were two-sided, *P* < 0.05 was considered statistically significant.

## Results

A total of 29,516 participants (13,410 males, and 16,106 females) were included in this study with a mean age of 56.48 ± 13.13 years. The overall weighted prevalence of CKD was 3.94% (3.62% in male and 4.25% in female).

Table 1 presents the characteristics of individuals included in the current analysis. The prevalence of CKD in both male and female tended to increase with age (*P* for trend < 0.001). Furthermore, we found that participants who had hypertension, diabetes mellitus, myocardial infarction or stroke had a higher risk of CKD in both genders (all *P* < 0.001). In addition, the values of BMI, WC, WHtR, PBF, and VFI almost consistently higher among participants with CKD, while only WHtR, PBF and VFI had statistical significant in both genders (all *P* < 0.001).

**Table 1:** Baseline characteristics of study population

	Male (*n* = 13,410)			Female (*n* = 16,106)			Total (*n* = 29,516)
	eGFR≥ 60	eGFR< 60	*P*	eGFR≥ 60	eGFR< 60	*P*	
Age (year)							
35~ 44	2942 (23.11)	17 (2.49)	< 0.0001	3953 (25.99)	23 (2.57)	< 0.0001	6935 (23.50)
45~ 54	3001 (23.58)	40 (5.87)		3950 (25.97)	61 (6.82)		7052 (23.89)
55~ 64	2938 (23.08)	95 (13.93)		3453 (22.70)	144 (16.11)		6630 (22.46)
65~ 74	2476 (19.45)	237 (34.75)		2566 (16.87)	295 (33.00)		5574 (18.88)
≥ 75	1371 (10.77)	293 (42.96)		1290 (8.48)	371 (41.50)		3325 (11.27)
Urban	5870 (46.12)	305 (44.72)	0.4760	6636 (43.62)	405 (45.30)	0.3250	13,216 (44.78)
Region							
Eastern	5464 (42.93)	302 (44.28)	< 0.0001	6549 (43.05)	339 (37.92)	< 0.0001	12,654 (42.87)
Middle	5200 (40.85)	220 (32.26)		5814 (38.22)	283 (31.66)		11,517 (39.02)
Western	2064 (16.22)	160 (23.46)		2849 (18.73)	272 (30.43)		5345 (18.11)
Higher Education (> 12 years)	957 (7.52)	46 (6.74)	0.4540	847 (5.57)	24 (2.68)	< 0.0001	1874 (6.35)
Married	11,640 (91.47)	547 (80.21)	< 0.0001	13,263 (87.19)	565 (63.20)	< 0.0001	26,015 (88.14)
Smoking status	5861 (46.05)	226 (33.14)	< 0.0001	516 (3.39)	42 (4.70)	0.0380	6645 (22.51)
Alcohol use	4946 (38.86)	157 (23.02)	< 0.0001	541 (3.56)	28 (3.13)	0.5040	5672 (19.22)
Hypertension	5184 (40.73)	440 (64.52)	< 0.0001	5603 (36.83)	589 (65.88)	< 0.0001	11,816 (40.03)
DM	1281 (10.06)	114 (16.72)	< 0.0001	1298 (8.53)	152 (17.00)	< 0.0001	2845 (9.64)
MI	97 (0.76)	17 (2.49)	< 0.0001	93 (0.61)	15 (1.68)	< 0.0001	222 (0.75)
Stroke	340 (2.67)	54 (7.92)	< 0.0001	266 (1.75)	40 (4.47)	< 0.0001	700 (2.37)
Weight (kg)	67.61 ± 11.19	65.65 ± 11.90	< 0.0001	59.45 ± 9.99	57.83 ± 10.78	< 0.0001	63.05 ± 11.36
BMI (kg/m^2^)	24.40 ± 3.36	24.28 ± 3.64	0.3585	24.70 ± 3.64	24.80 ± 4.03	0.4103	24.56 ± 3.53
WC (cm)	85.43 ± 9.93	85.73 ± 10.77	0.4479	82.31 ± 10.04	84.75 ± 11.34	< 0.0001	83.81 ± 10.17
WHtR	0.51 ± 0.06	0.52 ± 0.06	0.0003	0.53 ± 0.07	0.56 ± 0.07	< 0.0001	0.52 ± 0.06
PBF	24.92 ± 5.34	26.59 ± 5.51	< 0.0001	33.25 ± 5.23	35.16 ± 5.78	< 0.0001	29.56 ± 6.75
VFI	11.04 ± 4.79	12.02 ± 5.59	< 0.0001	7.87 ± 4.08	9.12 ± 4.86	< 0.0001	9.37 ± 4.74

*eGFR* estimated glomerular filtration rate, the unit was as ml/min/1.73m^2^, *BMI* body mass index, *WC* waist circumference, *WHtR* waist-to-height ratio, *PBF* percent body fat, *VFI* visceral fat index, *DM* diabetes mellitus, *MI* myocardial infarction

Data are expressed as the mean ± SD or as n (%)

Pearson’s correlation coefficients for the 5 adiposity indices each against eGFR stratified by gender are illustrated in Table 2. We found that all these indices had a significant negative correlation to eGFR (*P* < 0.0001). BMI had the lowest Pearson’s correlation coefficient in both genders (Pearson’s correlation coefficient:− 0.051 in males; − 0.014 in females) which indicated the weakest correlations with eGFR.

**Table 2:** Pearson’s correlation coefficients between adiposity indices and eGFR

	eGFR			
	Male	*P*	Female	*P*
BMI (kg/m^2^)	-0.051	< 0.0001	−0.014	< 0.0001
WC (cm)	−0.061	< 0.0001	−0.070	< 0.0001
WHtR	−0.069	< 0.0001	−0.097	< 0.0001
PBF	−0.129	< 0.0001	− 0.151	< 0.0001
VFI	−0.139	< 0.0001	−0.112	< 0.0001

*eGFR* estimated glomerular filtration rate, *BMI* body mass index, *WC* waist circumference, *WHtR* waist-to-height ratio, *PBF* percent body fat, *VFI* visceral fat index

**Fig. 1:**
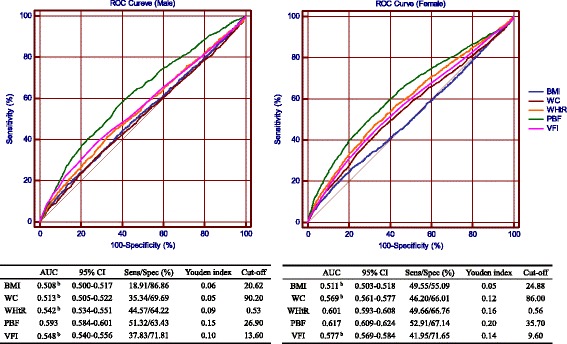
AUC of adiposity indices to identifying subjects with chronic kidney disease according to sex. Sens: sensitivity; Spec: specificity; ROC curve: receiver operating characteristics curve; AUC: area under the ROC curve; BMI: body mass index; WC: waist circumference; WHtR: waist-to-height ratio; PBF: percentage body fat; VFI: visceral fat index

Figure 1 shows the AUC values (95% confidence interval (CI)) and Youden index for each of the five adiposity indices in prediction of CKD. PBF had a significantly higher AUC in both male and female groups (AUC for males: 0.593, 95% CI: 0.584–0.601; AUC for females: 0.617, 95% CI: 0.609–0.624) than the other indices (with WHtR in females being an exception). PBF also yielded the highest Youden index in identifying CKD (male: 0.15; female: 0.20).

Odds ratios (ORs) for the association between the CKD and high levels of various adiposity indices stratified by gender are shown in Table 3. PBF had the highest crude OR for CKD in male [1.819 (1.559–2.123)] and in female groups [2.268 (1.980–2.597)]. After adjusted for confounding factors, the ORs on PBF were also significant for both genders (*P* < 0.01). Table 4 shows the performance of the five adiposity indices according to Z-score standardization stratified by gender. As shown, after adjusted for confounding factors, a 1-SD increment change for each adiposity index was associated with higher risk of CKD in both male and female (all *P* < 0.05). PBF had the highest ORs for CKD in both genders.

**Table 3:** Odds ratios and 95% confidence interval for chronic kidney disease according to adiposity indices stratified by gender

	Model 1	Model 2	Model 3
Male			
BMI	0.650 (0.533–0.792)^b^	1.061 (0.857–1.314)	0.954 (0.768–1.186)
WC	1.248 (1.062–1.466)^b^	1.617 (1.355–1.929)^b^	1.445 (1.205–1.731)^b^
WHtR	1.357 (1.163–1.584)^b^	1.446 (1.225–1.708)^b^	1.295 (1.092–1.535)^b^
PBF	1.819 (1.559–2.123)^b^	1.728 (1.476–2.024)^b^	1.409 (1.197–1.657)^b^
VIF	1.549 (1.321–1.817)^b^	1.519 (1.279–1.804)^b^	1.357 (1.138–1.617)^b^
Female			
BMI	1.196 (1.045–1.368)^b^	1.434 (1.239–1.660)^b^	1.301 (1.121–1.511)^b^
WC	1.647 (1.439–1.886)^b^	1.390 (1.202–1.609)^b^	1.262 (1.087–1.464)^b^
WHtR	1.961 (1.713–2.246)^b^	1.303 (1.127–1.507)^b^	1.188 (1.025–1.378)^a^
PBF	2.268 (1.980–2.597)^b^	1.956 (1.701–2.249)^b^	1.520 (1.315–1.757)^b^
VIF	1.824 (1.589–2.092)^b^	1.422 (1.227–1.648)^b^	1.284 (1.105–1.493)^b^

*BMI* body mass index, *WC* waist circumference, *WHtR* waist-to-height ratio, *PBF* percent body fat, *VFI* visceral fat index

Data are expressed as the odds ratios (95% confidence interval)

Model 1: Non-adjusted

Model 2: Adjusted for age, smoking status, alcohol use, education level, marital status, living area (rural/urban) and region (eastern, middle and western)

Model 3: Adjusted for age, smoking status, alcohol use, education level, marital status, living area (rural/urban), region (eastern, middle and western), hypertension, diabetes mellitus, myocardial infarction and stroke

^a^*P* < 0.05, ^b^*P* < 0.01

**Table 4:** Standardized odds ratios and 95% confidence interval for chronic kidney disease according to adiposity indices stratified by gender

	Model 1	Model 2	Model 3
Male			
BMI z-score	0.964 (0.892–1.042)	1.241 (1.143–1.347)^b^	1.163 (1.068–1.266)^b^
WC z-score	1.030 (0.954–1.113)	1.203 (1.109–1.305)^b^	1.128 (1.037–1.226)^b^
WHtR z-score	1.153 (1.067–1.245)^b^	1.175 (1.085–1.272)^b^	1.103 (1.016–1.197)^a^
PBF z-score	1.376 (1.272–1.488)^b^	1.329 (1.227–1.439)^b^	1.186 (1.091–1.289) ^b^
VIF z-score	1.216 (1.129–1.309)^b^	1.215 (1.127–1.309)^b^	1.145 (1.059–1.237)^b^
Female			
BMI z-score	1.029 (0.962–1.100)	1.170 (1.092–1.253)^b^	1.108 (1.032–1.190)^b^
WC z-score	1.267 (1.185–1.354)^b^	1.194 (1.116–1.278)^b^	1.138 (1.062–1.220)^b^
WHtR z-score	1.423 (1.332–1.519)^b^	1.146 (1.071–1.227)^b^	1.093 (1.020–1.172)^a^
PBFz-score	1.485 (1.380–1.599)^b^	1.337 (1.242–1.441)^b^	1.153 (1.070–1.244)^b^
VIF z-score	1.302 (1.227–1.381)^b^	1.167 (1.094–1.244)^b^	1.108 (1.036–1.185)^b^

*BMI* body mass index, *WC* waist circumference, *WHtR* waist-to-height ratio, *PBF* percent body fat, *VFI* visceral fat index

Data are expressed as the odds ratios (95% confidence interval)

Model 1: Non-adjusted

Model 2: Adjusted for age, smoking status, alcohol use, education level, marital status, living area (rural/urban) and region (Eastern, middle and Western)

Model 3: Adjusted for age, smoking status, alcohol use, education level, marital status, living area (rural/urban), region (Eastern, middle and Western), hypertension, diabetes mellitus, myocardial infarction and stroke

^a^*P* < 0.05, ^b^*P* < 0.01

## Discussion

The prevalence of CKD (eGFR< 60 ml/min/1.73m^2^) in middle-aged Chinese adults was 3.94% (3.62% in male and 4.25% in female). All of the adiposity indices (BMI, WC, WHtR, PBF and VFI) had a significant negative correlation to CKD in both genders. Obesity is associated with an increased risk of CKD. For the comparison, we found that PBF was a more suitable screening tool for predicting CKD than other adiposity indices for CKD in both males and females.

Several cross-sectional studies demonstrated that the prevalence of CKD differed substantially between geographic regions in China. The prevalence of CKD (eGFR< 60 ml/min/1.73m^2^) in southern China was 3.2% (2.2% in male vs. 4.1% in female) [16] which was a little lower than the results in our study. While, in the study conducted in Beijing among participants older than 40 years found that the prevalence of reduced renal function (eGFR< 60 ml/min/1.73m^2^) was 5.2% [17], which was higher than our present study. In addition to methodology, the heterogeneity might be related to differences in lifestyles, medical care and socioeconomic characteristics in different regions of China [18].

Previously, the majority of existing literature has focused on the relationship between BMI and/or WC with CKD. A cross-sectional study conducted in Chinese adults showed that for each increase of 1.0 kg/m^2^ in BMI, a decline of 0.5 ml/min/1.73m^2^ [19]. In addition, a prospective cohort study, which included 827 incident cases of ESRD occurred during an average follow-up of 15.5 years, showed that patients had more than 1.1 times higher risk for developing ESRD participants with a higher BMI (BMI ≥ 27.5 kg/m^2^), compared to a normal BMI (18.5 to 23 kg/m^2^) [20]. Furthermore, a 7-year population-based cohort study conducted in Iran indicated that, irrespective of general obesity, the risk of developing CKD rose with increasing WC [21]. Even though BMI and WC have been the most widely used measurements to classify obesity, BMI does not account for fat distribution [22]; and WC does not account for height [11]. Thus, other indices which can more accurately measure total body fat quantity and visceral fat, such as WHtR, PBF and VFI, were developed. In our study, we found that WHtR, PBF and VFI correlated much more strongly with eGFR than BMI and WC (according to Pearson’s correlation coefficients in Table 2).

A related study conducted among female subjects aged 65–80 years found that WHtR was a better index associated with CKD when comparing with other common adiposity indices (BMI, WC, waist-to-hip ratio) [23]. Dai et al. suggested that a visceral adiposity index was superior to BMI and WC for predicting CKD in females [24]. Additionally, a cross-sectional study [25] conducted in Chinese adults indicated that body fat percentage was significantly related to increased risk of CKD (OR: 1.049, 95%CI: 1.006–1.093). Our study found that PBF had the highest AUC in both male and female groups. After adjusting for confounding factors, PBF still had the highest ORs for CKD than other adiposity indices in both genders.

There are a few mechanisms that can explain the association between obesity and renal injury, including hemodynamic effects, inflammation and renal lipotoxicity [26]. Angiotensin II is elevated in the blood of obese individuals, leading to vasoconstriction of efferent arterioles; plasma aldosterone is also slightly elevated in obese individuals, causing salt and water retention, and ultimately proteinuria [26, 27]. In regarding to inflammation, cytokines such as interleukin-6 (IL-6) and tumor necrosis factor – alpha (TNF-α) play a major role in CKD development. IL-6 invades the adipose tissue and influences transforming growth factor – beta 1 (TGF-β1) receptor trafficking, leading to renal fibrosis [28]. TNF-α inhibits the activity of the nephron gene promoter in cultured podocytes, resulting in podocyte dysfunction in the obese population [29, 30].

Our study has several strengths. Firstly, we enrolled a large population-based sample, including male and female participants from China. Secondly, to our knowledge, this is the first time a comparison across five adiposity indices (BMI, WC, WHtR, PBF, VFI) has been made to predicting CKD in a large Chinese population. However, there are several potential limitations that should be considered when interpreting the results. First, this is a cross-sectional study and no causal relationship can be established. Further follow-up of the population and more prospective studies will be needed to validate our findings. Secondly, some variables such as physical activity and dietary patterns which might affect renal function were not included in our study. Finally, the gold standards for measurement of fat distribution are micromagnetic resonance imaging and microcomputed tomography [31]; in our investigation, we used the Omron body composition monitor, which may be less accurate than gold standards. However, the Omron device has been used in various studies and has demonstrated reasonable accuracy [32, 33].

## Conclusion

Obesity was associated with an increased risk of CKD, with PBF being the best adiposity index predictor for identifying CKD. Given that obesity is modifiable risk factor, CKD patients should manage their weight to prevent CKD progression. Patients should also be screened for high PBF in CKD prevention efforts.

## Additional files

Additional file 1: List of investigators of the China Hypertension Survey Study. (DOCX 14 kb)

Additional file 2: List of IRBs of sub-centers. (DOCX 13 kb)

## Abbreviations

AUC: Area under the ROC curve
BMI: Body mass index
CKD: Chronic kidney disease
eGFR: Estimated glomerular filtration rate
PBF: Percentage body fat
ROC curve: Receiver operating characteristics curve
VFI: Visceral fat index
WC: Waist circumference
WHtR: Waist-to-height ratio
