# Supplementary material for: Comparison of visceral, body fat indices and anthropometric measures in relation to chronic kidney disease among Chinese adults from a large scale cross-sectional study
Source: BMC Nephrol. 2018 Feb 17;19:40. doi: 10.1186/s12882-018-0837-1 (PMC5816526; doi:10.1186/s12882-018-0837-1)
Supplement: Supplementary file 1 — List of investigators of the China Hypertension Survey Study. (DOCX 14 kb) [file 12882_2018_837_MOESM1_ESM.docx]

**Appendix 1: List of investigators of the China Hypertension Survey Study**

This study was accomplished through the fine work of the staff at the national level. For a partial listing of colleagues see the follows (provinces sorted as alphabetical order):

**Anhui**: Anhui Provincial Hospital (Liqun Hu, Hongqi Li, Qi Zhang, Guang Yan); Anhui Institute of Cardiovascular Disease (Fangfang Zhu).

**Beijing**: Xuanwu Hospital, Capital Medical University (Xianghua Fang, Chunxiu Wang, Shaochen Guan, Xiaoguang Wu, Hongjun Liu, ChengbeiHou).

**Chongqing**: First Affiliated Hospital of Chongqing Medical University (Han Lei, Wei Huang, Nan Zhang); Chongqing Medical University (Ge Li, Lihong Mu, Xiaojun Tang).

**Gansu**: Lanzhou University Second Hospital (Jing Yu, Xiaowei Zhang, Wei Liang, Heng Yu, Qiongying Wang); Maternal and Child Care Service Centre (Lan Yang).

**Guangxi**: First Affiliated Hospital of Guangxi Medical University (Tangwei Liu, Rongjie Huang, Zhiyuan Jiang, Haichan Qin).

**Hebei**: Center for Disease Prevention and Control of Hebei (Jixin Sun, Yajing Cao, Yuhuan Liu); Center for Disease Prevention and Control of Tangshan (Zhikun Zhang); Center for Disease Prevention and Control of Langfang (Yanmei Liu); Center for Disease Prevention and Control of Xingtai (Dejin Dong); Center for Disease Prevention and Control of Dingzhou (Guangrong Li).

**Heilongjiang**: First Affiliated Hospital of Harbin Medical University (Hong Guo, Lihang Dong, Haiyu Zhang, Fengyu Sun, Xingbo Gu, Ye Tian).

**Henan**: Zhengzhou University (Kaijuan Wang, Chunhua Song, Peng Wang, Hua Ye); Henan Academy of Medical Sciences (Wei Nie, Shuying Liang).

**Hubei**: Department of Cardiology, Renmin Hospital of Wuhan University (Congxin Huang, Fang Chen, Yan Zhang, Heng Zhou, Jing Xie, Jianfang Liu).

**Jiangsu**: First Affiliated Hospital of Nanjing Medical University (Xiangqing Kong, Ming Gui, Wenhua Xu, Yan Lu, Jun Huang); Affiliated Hospital of Nantong University (Min Pan); Center for Disease Control and Prevention of Jiangsu (Jinyi Zhou).

**Liaoning**: Center for Disease Prevention and Control of Liaoning (Jun Na, Li Liu, Yanxia Li, Guowei Pan); Health and Family Planning Commission of Liaoning (Degang Dong, Peng Qu).

**Qing Hai**: Qing Hai Center for Disease Control and Prevention (Jianning Yue, Minru Zhou, Zhihua Xu, Xiaoping Li, Qiongyue Sha, Fuchang Ma); Qinghai Cardio-Cerebrovascular Disease Special Hospital (Qiuhong Chen, HuipingBian).

**Shanxi**: Shanxi Cardiovascular Hospital (Bao Li, Lijun Zhu, Yuean Zhang, Gang Wang); Wuxiang County People's Hospital (Zhihan Hao).

**Zhejiang**: Zhejiang Hospital (Xinhua Tang, Jing Yan, Xiaoling Xu, Li Yang, Aimin Jiang, Wei Yu).
