# Supplementary material for: Comparison of visceral, body fat indices and anthropometric measures in relation to chronic kidney disease among Chinese adults from a large scale cross-sectional study
Source: BMC Nephrol. 2018 Feb 17;19:40. doi: 10.1186/s12882-018-0837-1 (PMC5816526; doi:10.1186/s12882-018-0837-1)
Supplement: Supplementary file 2 — List of IRBs of sub-centers. (DOCX 13 kb) [file 12882_2018_837_MOESM2_ESM.docx]

**Appendix 2: List of IRBs of sub-centers**

Anhui Provincial Hospital, Anhui Institute of Cardiovascular Disease, Anhui, China;

Xuanwu Hospital, Capital Medical University, Beijing, China;

First Affiliated Hospital of Chongqing Medical University, Chongqing Medical University, Chongqing, China;

Lanzhou University Second Hospital, Gansu, China;

First Affiliated Hospital of Guangxi Medical University, Guangxi, China;

Center for Disease Prevention and Control of Hebei, Hebei, China;

First Affiliated Hospital of Harbin Medical University, Helongjiang, China;

Zhengzhou University; Henan Academy of Medical Sciences, Henan, China;

Renmin Hospital of Wuhan University, Hubei, China;

First Affiliated Hospital of Nanjing Medical University, Jiangsu, China;

Center for Disease Prevention and Control of Liaoning, Liaoning, China;

Qing Hai Center for Disease Control and Prevention, Qinghai, China;

Shanxi Cardiovascular Hospital, Shanxi, China;

Zhejiang Hospital, Zhejiang, China.
